# Automated recognition of the cricket batting backlift technique in video footage using deep learning architectures

**DOI:** 10.1038/s41598-022-05966-6

**Published:** 2022-02-03

**Authors:** Tevin Moodley, Dustin van der Haar, Habib Noorbhai

**Affiliations:** 1 Academy of Computer Science and Software Engineering, University of Johannesburg, Cnr University Road and Kingsway Avenue, Auckland Park Johannesburg, 2092 Gauteng, South Africa; 2 Biomedical Engineering and Healthcare Technology (BEAHT) Research Centre, Faculty of Health Sciences, University of Johannesburg, Cnr Siemert Beit Street, Doornfontein City Two, 2028 Gauteng, South Africa

**Keywords:** Image processing, Machine learning

## Abstract

There have been limited studies demonstrating the validation of batting techniques in cricket using machine learning. This study demonstrates how the batting backlift technique in cricket can be automatically recognised in video footage and compares the performance of popular deep learning architectures, namely, AlexNet, Inception V3, Inception Resnet V2, and Xception. A dataset is created containing the lateral and straight backlift classes and assessed according to standard machine learning metrics. The architectures had similar performance with one false positive in the lateral class and a precision score of 100%, along with a recall score of 95%, and an f1-score of 98% for each architecture, respectively. The AlexNet architecture performed the worst out of the four architectures as it incorrectly classified four images that were supposed to be in the straight class. The architecture that is best suited for the problem domain is the Xception architecture with a loss of 0.03 and 98.2.5% accuracy, thus demonstrating its capability in differentiating between lateral and straight backlifts. This study provides a way forward in the automatic recognition of player patterns and motion capture, making it less challenging for sports scientists, biomechanists and video analysts working in the field.

## Introduction

Cricket batting has evolved considerably in recent years, with an added emphasis on the shorter formats of the game^1^. There has been a growing need to maximise performance and success at the highest level, as well as to understand particular playing patterns through sophisticated analysis and machine learning^2^.

The increased use of technology, combined with science and medicine, has been labelled as a game-changer within the sporting domain, with an emphasis on analysis^3^. The technological growth has seen significant breakthroughs within sports video content analysis, particularly through the advances in artificial intelligence, deep learning and multimedia technologies. Sports video analysis is domain dependant with unique challenges, which identifies several areas of research that require further investigation^4,5^. Several studies have exploited the use of technology where one particular study attempts to measure the vertical jump performance reliably using an iPhone application called *My Jump*^6^. Several other mobile applications have been developed to assist in player performance and provide feedback from a biomechanics perspective^7–9^. In the cricketing domain, mobile applications have been developed to analyse team performance, player injury, and match prediction^3,10–12^. While these applications have made several improvements within the cricketing domain, there is a lack of research dedicated toward the enhancement and improvement of cricket batting^13^.

The cricket batting technique is intricate that involves a series of complex gestures needed to perform a stroke, one of these gestures performed by the batsman is referred to as the batting backlift technique (BBT)^14^. Previous research has indicated that the BBT can be seen as a contributing factor to successful batsmanship^13,15,16^. There are two backlifts investigated in this study, namely the *lateral* batting backlift technique (LBBT), and the *straight* batting backlift technique (SBBT). The LBBT is a technique present where the toe and face of the bat are lifted laterally in the direction of second slip. The SBBT is represented whenever the toe and face of the bat are pointed toward the stumps and ground^13^.

### Related works

Conventionally, many video analysts would record footage using video cameras and link these to their parent analysis software to identify player performances, patterns, as well as kinetic and kinematic analyses. However, at times, such processes can be a tedious task. In the age of the fourth industrial revolution where automation and deep learning can be utilised to enhance real-time analysis and various identifications of match play, this provided impetus for further studies to document such validations.

There have been limited studies conducted demonstrating the validation of batting techniques in cricket either through mobile applications, platforms, machine learning or artificial intelligence. There has been an increase in applying computer vision techniques within the context of cricket. One example^17^ propose a cricket stroke recognition model, which demonstrates how various cricket strokes such as block cut, drive and glance in cricket can be automatically recognised in video footage using different traditional and deep learning architectures.

A study conducted by^18^ propose a model that uses a deep CNN to recognise cricket batting shots. The proposed method is based on 2D convolution followed by a recurrent neural network for processing sequence of video frames and a 3D convolution network for capturing spatial and temporal features simultaneously^18^. The dataset used comprised of 800 batting shot clips consisting of the drive, pull, hook, cut, sweep and flick strokes. The model is able to recognise the different strokes being performed with 90% accuracy. The high model accuracy noted the implications of modern deep learning in applications for detecting various cricket activities and for decision making.

A cricket shot detection model is presented by^19^, which proposes a novel scheme to recognise various cricketing strokes. The proposed model uses a deep convolutional neural network that relies on saliency and optical flow to highlight static and dynamic cues. The study proposes an entirely new dataset consisting of 429 video clips of the different types of drive strokes performed by a batsman^19^. The proposed framework achieves an accuracy of 97.69% for a left-handed batsman and 93.18% for a right-handed batsman. The authors noted that future work would focus on extending the incorporation of native features for every defined stroke.

In each of the studies highlighted, there has been an attempt to recognise different cricketing strokes using deep learning methods on entirely different datasets. These studies highlight the success of deep learning within the problem domain and further motivate the use of deep learning within the cricketing context. While deep learning has successfully recognised different cricket strokes, there are limited studies and no datasets that address the cricketing backlift, which is a key aspect of the batting technique. By applying deep learning methods to the backlift recognition task, this research is able to achieve automated recognition of the cricket batting backlift technique in video footage, which is novel within the cricketing environment. Additionally, the dataset created in this study can be used upon request, which will benchmark future works.

The contributions of the article can be noted as follows; producing a model to achieve an end to end backlift recognition, the use of transfer learning and how it improves performance in the context even in the presence of little data, the Xception architecture is the best performing architecture for the backlift recognition task, which in itself is a largely unexplored area, and finally, the dataset produced the firsts of its kind, which will allow future works to be implemented using a baseline dataset.

The outline of this study begins with section “Methods”, which unpacks the terminology, the dataset used and the different types of architectures implemented in this study. Section “Results” discusses the results obtained in the study, which is further unpacked in section “Discussion”. Finally, the contributions and future works are outlined in section “Conclusion”.

## Methods

Traditionally, types of backlift (lateral or straight) are categorised manually using an expert and analysing specific pose and alignment attributes. In this study, we attempt to automate this process by following deep learning-based methods instead of using a hand-crafted feature engineered based approach. It uses representational learning that implicitly constructs its own features to differentiate between the types of backlift by training on known cases of each backlift class.

We start by first creating a dataset of images that contains two types of backifts, along with annotations specifying which type of backlift each image depicts. We then select, create and train deep learning architectures using the created dataset and benchmark against common machine learning metrics to determine its efficacy.

### Dataset creation

There are specific requirements that must be considered during the construction of the dataset. The process began through a comprehensive YouTube search of First-Class International Cricket Test Match highlights, where the match’s environment has fewer variations to consider. The investigation was to select various batsmen who performed the lateral and straight backlifts, ten batsmen that exhibited the straight backlift and ten batsmen that demonstrated the lateral backlift. The batsman selected for the straight backlift; Babar Azam, Themba Bavuma, Rahul Dravid, JP Duminy, Dean Elgar, Mahela Jayawardene, Ajinkya Rahane, Joe Root, Rory Burns, Ben Stokes, and David Warner. The batsmen selected for the lateral backlift; AB de Villiers, Hashim Amla, Quintin de Kock, Faf du Plessis, Kevin Pieterson, Kumar Sangakara, Brian Lara, Ricky Ponting, Steve Smith, and Virat Koli. Using the Labelbox editor, each object within the scene is labelled, allowing for easier isolation and extraction of the batsman in each frame. The frame used for constructing the dataset was when the bowler is about to release the ball towards the batsman. The frame is identified as the ideal time period for the position of the batsman at the instant of delivery^20^. Using an 80:20 data split, the training class had 160 images, and the testing class had 40 images, resulting in a total of 200 images, which will serve as a baseline to draw comparisons of the proposed architectures. The image aspect ratio chosen through testing and validation is $128\times 128$, which is chosen to avoid distorting the original image.

### Model for implementation

The models proposed for this research paper is the AlexNet, Inception V3, Inception Resnet V2, and Xception architecture^21^. As of late, deep learning has gathered tremendous success in various domains^22,23^. The AlexNet architecture, which is relatively older, belongs to the deep Convolutional Neural Network (CNN) structure proposed by Krizhevsky and subsequently won the ImageNet object recognition challenge in 2012^24^.

**Figure 1:**
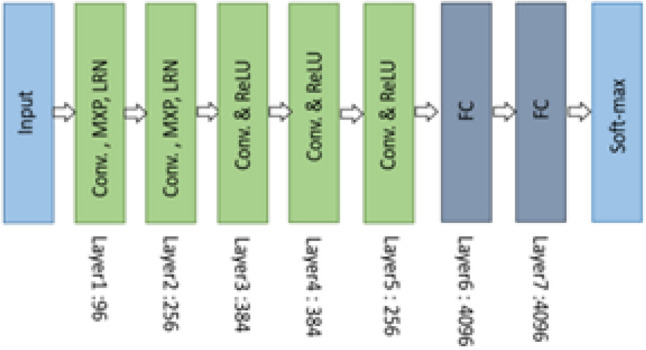
The AlexNet architecture depicting the different layers used for classification^22^.

AlexNet managed to achieve recognition accuracy that was better than most traditional machine learning approaches of the time^22^. The significant breakthrough in machine learning and computer vision-related tasks has seen the AlexNet architecture widely used across various domains^25^. Figure 1, depicts the structure of the AlexNet architecture. Local Response Normalisation (LRN) is performed at the first layer using 96 receptive filter^22^, where LRN is responsible for connecting the layers using an organised spatial pattern. Max pooling allows for dimensionality reduction in which assumptions are subsequently derived concerning the features contained within the sub-regions and is performed using $3\times 3$ filters with a size of 2. Each layer has a number of kernels with a specific size. The second ($5\times 5\times 48$) layer has 256, the third ($3\times 3\times 256$) and fourth ($3\times 3\times 192$) have 384, and the fifth ($3\times 3\times 192$) has 256 kernels^23^. Layer six and seven is the fully-connected layers made up of 4096 neurons each. Finally, the softmax fully-connected layer represents the number of labelled classes.

In 2014, Szegedy et al., introduced a newer network known as GoogleLeNet, otherwise known as Inception V1. The Inception V1 architecture was then refined over the years and subsequently referred to as Inception V2, Inception V3, and Inception Resnet^26^. The Inception V3 model was proposed that consisted of 48 layers. Unlike the AlexNet architecture, the Inception V3 architecture performs some of its calculations simultaneously^27^. Szegedy et al. describe the Inception V3 architecture^28^. The traditional $7\times 7$ convolution is factorised into three $3\times 3$ convolutions. The inception part of the network has three traditional modules at the $35\times 35$ convolution with 288 filters each, which is reduced to a $17\times 17$ grid with 7968 filters^28^. The inception modules are then followed by five instances of factorised inception modules, which is reduced to an $8\times 8\times 1280$ grid using the grid reduction technique.

**Figure 2:**
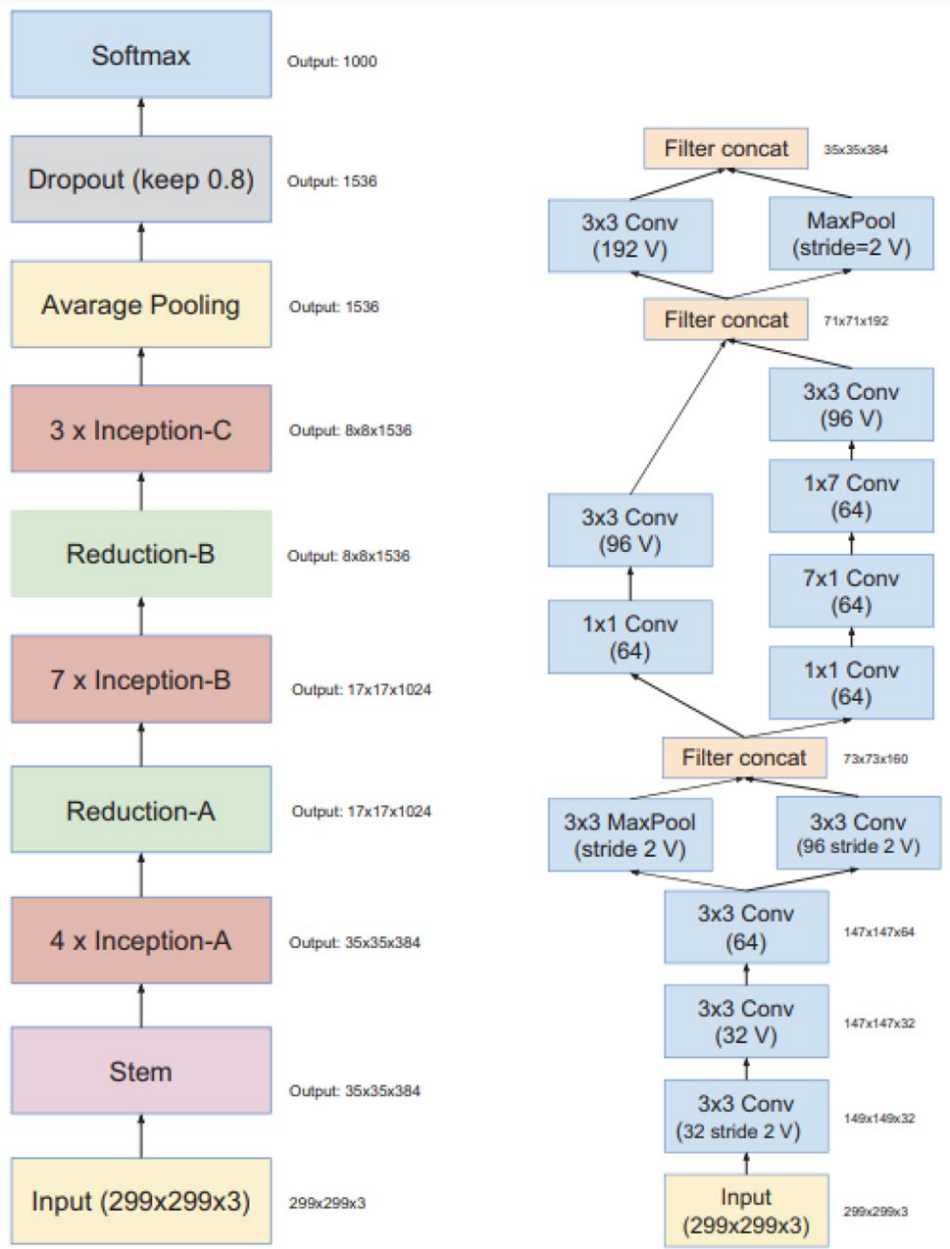
The left side represents the overall schema for the pure Inception Resnet V2, where the right side illustrates the detailed composition of the stem^30^.

Using the Inception architecture and residual connections, the Inception Resnet V2 architecture was formed^29^. Inception Resnet V2 is a convolutional neural network that implements concatenation in each multi-branch architecture. Residual models are well known for training very deep architectures^29^. Figure 2 represents the overall schema and the detailed composition of the Inception Resnet V2 architecture, where the inception blocks can be seen^30^. Using the filter expansion layer and residual modules after each inception block, the dimensionality of the filter bank is scaled up to compensate for the dimensionality reduction induced by the inception block^30^.

**Figure 3:**
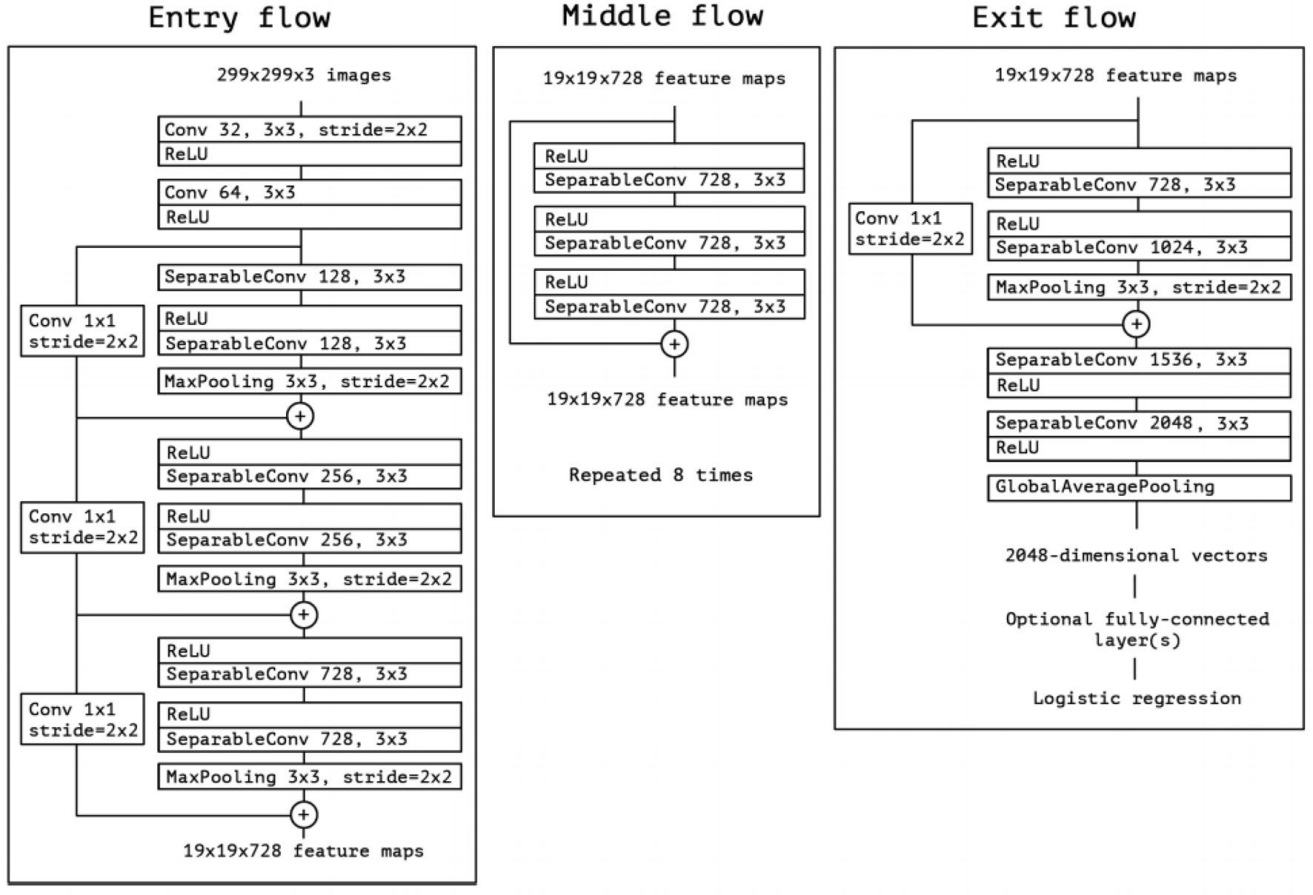
A figure representing the Xception architecture^26^.

According to Chollet, the architecture dubbed as Xception is said to slightly outperform the Inception V3 architecture on the ImageNet dataset^26^. The Xception architecture is made up of 36 convolution layers and is able to decouple the mapping between cross-channel correlations and spatial correlations. Each layer is structured into 14 modules, where each module has a linear residual connection around it^26^. Figure 3 represents the Xception architecture that undergoes an entry flow, middle flow, and exit flow.

Transfer learning allows for the transfer of knowledge from one task to another, which has been used in highly specialised disciplines, where the availability of large scale quality data proves challenging^31^. Generally, a neural network requires a large dataset to train from scratch, unfortunately, these large datasets are not always available, which is why transfer learning is beneficial in this study, where the dataset is noted for being smaller in size. Transfer learning draws a starting point in which a good machine learning model can be built with comparatively little training data since the model is already pre-trained. Pre-trained models are seen as the efficient optimisation procedure, which also supports the improvement of classification problems^31^. In order to justify the architecture of choice, Table 1 is added that compares the top-1 and top-5 accuracy of the chosen architectures in the study. Using the top-1 and top-5 accuracy scores, the Inception V3, Inception Resnet V2, and Xception architectures have been compared on the ImageNet dataset, highlighting a performance comparison for each architecture. Top-1 accuracy refers to the conventional accuracy, where the model’s result must match the expected output exactly. Top-5 specifies any of the top 5 probabilities which must match the expected output. Table 1 illustrates a comparison of the various architectures on the ImageNet dataset. As seen in Table 1, the Xception architecture yields the best performance when applied to ImageNet with a top-1 accuracy of 80.3%, which serves as further evidence as to why the Xception architecture is the most suitable architecture for this study, further motivations will be unpacked in the results section.

**Table 1:** The Inception V3, Inception Resnet V2, and Xception architectures pre-trained and bench-marked on the ImageNet dataset to illustrate each architectures performance on a generalised dataset and to justify the selection of architectures in the study.

Network	Top-1 Accuracy	Top-5 Accuracy
Alexnet^22^	0.6330	0.8460
Inception V3^28^	0.790	0.945
Inception Resnet V2^30^	0.779	0.937
Xception^26^	0.803	0.953

While transfer learning offers many advantages related to time, computational complexity, accuracy, and smaller datasets, there are some limitations to its use^32^. One of the major challenges related to transfer learning is to produce positive transfer between tasks while avoiding negative transfer, which is a decrease in performance. Negative transfer occurs as there is no specific standard that defines the manner in which tasks are related from both domain and algorithmic perspectives, which makes it challenging to find solutions^32^. A more well-studied limitation surrounding transfer learning is overfitting that is apparent when a new model learns details and noises from training data that negatively impacts its output^32^. Fortunately, these limitations were not noted in this study. Overfitting was not encountered, and therefore, there was no need to reduce the network’s capacity, apply regularisation or add dropout layers.

During implementation, the following parameters were altered to ensure the Inception V3, Inception Resnet V2, and Xception architectures were fairly compared against the AlexNet architecture. Each architecture made use of transfer learning. For each architecture, the fully connected layer at the top of the network was set to *False*, which was done as the input shape of the images were changed to $128\times 128$ pixels. A global average pooling layer was added as a substitute fully connected layer. A dense output layer with activation softmax was added to match the number of classes in the study. The image weights were set to none. Again this is to ensure that the architectures are compared fairly to AlexNet. Finally, the input tensor is set to none, and the input size is set to $128\times 128$ pixels. No other parameters were altered.

Data augmentation techniques were applied to each architecture: shear range of 0.2, image rescaling of 1./255, horizontal flip set to true, and a zoom range of 0.2. Various parameters were selected through testing and validation; the activation parameter used is the rectified linear unit (ReLu), which manages to converge faster and more reliably in this study^33^. The softmax activation function is used at the output layer in order to predict a multinominal probability distribution. Using the Adam optimiser, the model manages the sparse gradients of noisy data. Using the various architectures defined in this study offers a novel approach to the problem domain, where metrics can determine the success of applying deep learning methods within the cricketing field. The results section will further unpack the findings of the proposed model and form a comparison on the different architectures to identify different areas of focus in future works. The source code may be found on the github repository, and the dataset may be used upon request.

### Data analysis

Each architecture’s performance is evaluated using the accuracy, confusion matrix, precision, recall, and f1-score metrics. To understand each metric the True Positives (TP), True Negatives (TN), False Positives (FP), and False Negatives (FN) values are defined. TP occurs when the proposed model manages to correctly predict positive observations, and TN occurs when the model correctly predicts negative observations. FP is apparent when the model incorrectly predicts positive observations, and the FN occurs when the model incorrectly predicts false observations. The model accuracy will formulate a comprehensive understanding of the architecture’s performance^34^. In order to testify to the effectiveness and efficiency of the results, multiple runs are carried out for each architecture. The results obtained in Table 2 is a result of running the data against each architecture ten times and computing the averages for each metric. Completing multiple runs ensures that the data is behaving correctly and the the results obtained are consistent verifiable across the respective architectures used in this study.

We can expect the model to successfully draw distinctions between the respective classes from the proposed architecture, thereby automatically recognising different backlifts in video footage, supported in the results section.

## Results

The metrics illustrated in Table 2 represents the confusion matrix for each architecture. The number of TP, FP, TN, and FN are highlighted. The AlexNet architecture struggles to correctly predict for the straight class, as it incorrectly predicts four images as a lateral backlift, where in fact, the images represent a straight backlift. The remaining architectures, Inception V3, Inception Resnet V2, and Xception, all have the same amount of misclassifications, each incorrectly predicting a single image as a straight backlift that represents a lateral backlift. The architectures accuracy and loss scores are illustrated in Table 2. The noteworthy observation is the loss score of the Xception architecture of 0.03%, which will be discussed in section 4.

**Table 2:** The confusion matrix representing each of the architectures across ten runs, where the false and true positive predictions for each class are represented.

Architecture	Class	Lateral	Straight	Accuracy (%)	Loss (%)
AlexNet	Lateral	19	1	82.45	34.17
	Straight	4	16		
Inception V3	Lateral	19	1	93.75	0.13
	Straight	0	20		
Inception Resnet V2	Lateral	19	1	96.1	0.12
	Straight	0	20		
Xception	Lateral	19	1	98.2	0.03
	Straight	0	20		

The metrics are shown in Table 3, which illustrates each architecture’s precision, recall, and f1-score for the respective classes. For each class (with a support of 20), the number of false positives is computed as shown in Table 2. Similarly to Table 2 the Inception V3, Inception Resnet V2, and Xception architectures all exhibit the same scores, the lateral class had a precision score of 100%, a recall score of 95%, and an f1-score of 97%. For the straight class, the three architectures had precision scores of 95%, recall scores of 100%, and f1-scores of 98%. The AlexNet architecture scores differed. The lateral class had a precision score of 83%, a recall score of 95%, and an f1-score of 88%. Finally, the straight class had a precision score of 90%, a recall score of 80%, and an f1-score of 86% as seen in Table 3.

**Table 3:** The average precision, recall, and f1-scores across ten runs for the lateral and straight backlifts for each architecture.

Architecture	Class	Precision (%)	Recall (%)	F1-Score (%)
AlexNet	Lateral	83	95	88
	Straight	94	80	86
Inception V3	Lateral	100	95	97
	Straight	95	100	98
Inception Resnet V2	Lateral	100	95	97
	Straight	95	100	98
Xception	Lateral	100	95	97
	Straight	95	100	98

## Discussion

**Figure 4:**
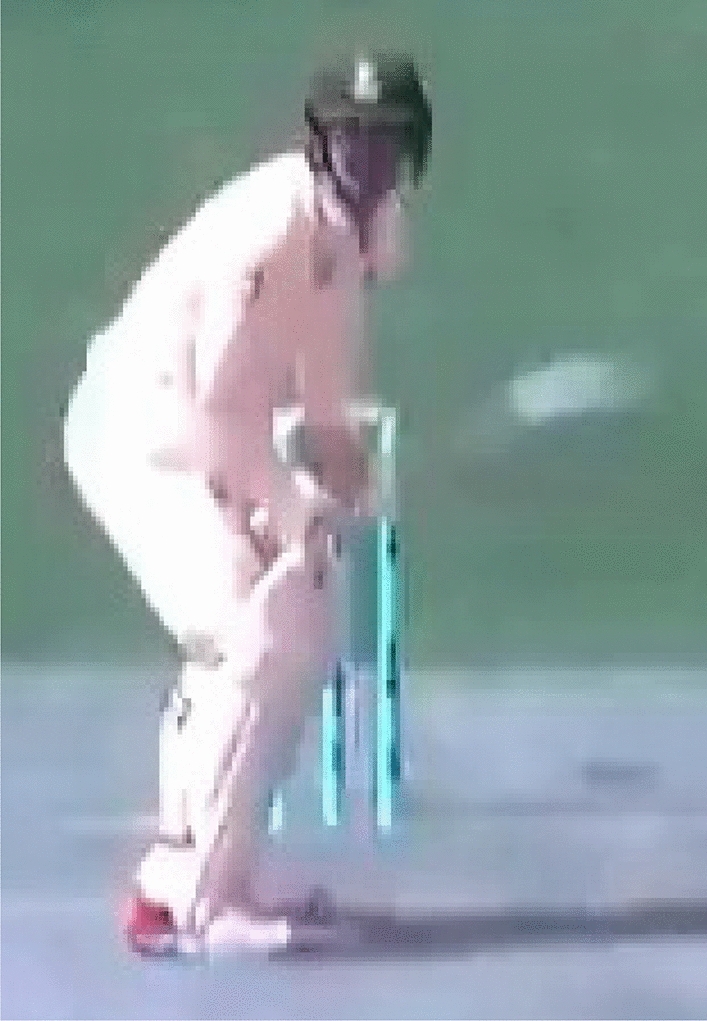
The image that is mispredicted by the Inception V3, Inception Resnet V2, and Xception architectures as a straight backlift, where the image represents a lateral backlift^35^.

This research paper looks to recognise different cricket backlifts using the AlexNet, Inception V3, Inception Resnet V2, and Xception architectures, which is successfully achieved as demonstrated by the results. The misclassifications highlighted in Table 2 for the Inception V3, Inception Resnet V2, and Xception architectures are inspected to determine if the misprediction is the same image across the board. The image shown in Fig. 4 is an example of an image that was misclassified for all three architectures, thus suggesting the image represents a high number of features that stem from a straight backlift. Further analysis of the respective image also highlights the difficulty to recognise the positioning of the bat. Due to the bat being one of the key determining factors of distinguishing between a lateral and straight backlift, it could be the reason for the misclassification. Reasons as to why the architectures make these false-positive predictions may be due to the bat being blurred, making it hard to determine the angle at which the bat is faced. However, the batsman involved is Quinton de Kock, who is known to have a lateral backlift.

The Inception V3, Inception Resnet V2 and Xception architectures have similar results making it difficult to determine which architecture would be best suited for the problem domain. At the same time, additional data in the future may provide a more concise and deeper knowledge. Each architecture within this study has been trailed and tested in a generalised context as seen in Table 1. These architectures have proven to be successful in various domains, contributing to advancements in computer vision. Traditionally the Xception architecture is said to yield better performance, which is largely due to its Top-1 accuracy^26^. The Top-1 accuracy measures the proportion of examples for which the predicted label matches the single target. The loss score of 0.05% for the Xception architecture further highlights the architectures ability to predict on a single image. It yielded the lowest lost score from all architectures coupled with the past performance Top-1 accuracy scores^26^, and it can be concluded that the Xception architecture is best suited for the problem domain. The performance of the Xception architecture further highlights the added benefit of transfer learning, the architecture both in a generalised context, as seen on the ImageNet dataset, and specialised context, as seen in this study, has outperformed the Inception V2 and Inception Resnet V2 architectures, thus highlighting its efficiency within the problem domain.

One study by^18^ identified and categorised various cricket batting shots from various videos. The approach and methods undertaken were based on deep convolutional neural networks. The first approach uses 2D CNN’s with recurrent neural networks for processing video footage. The second approach implements a 3D CNN to capture spatial and temporal features simultaneously. Using a dataset with approximately 800 batting shot clips, the proposed model achieved a 90% accuracy^18^. The high accuracy obtained in the study indicates the high implications of modern artificial intelligence and deep learning in applications for detecting various cricket activities and decision-making purposes. Similarly, in our study, the high accuracy was revealed to be 98.2%, further validating their argument.

A previous study by^13^ analysed the batting backlift technique using hand crafted features in the Open Computer Vision (CV) library, Android, and JavaScript. The system comprised of three main components; frontal view interface, lateral view interface, and a back-end system. The system was able to detect the type of backlift presented by the batsman by analysing and tracking the positional placement of batsman and the bat. The system was novel and provided and means to gather real-time data, which could be used for analysis. The improvements that deep learning has made within current research suggests that applications such as the one presented by^13^ can be improved drastically and requires further investigation.

Cust et al.^5^ postulated that “future work should look to adopt, adapt and expand on current models associated with a specific sports movement to work towards flexible models for mainstream analysis implementation. Investigation of deep learning methods in comparison to conventional machine learning algorithms will be of particular interest to evaluate if the trend of superior performances is beneficial for sport-specific movement recognition.” The approach demonstrated in this paper attempted to fill this void in which automation and automated recognition (coupled with deep learning methods) are key for player performance analysis in real-time.

## Conclusion

This research article was able to achieve an end to end backlift recognition, highlight the advantages of transfer learning, and identify the Xception architecture as the best performing architecture for the backlift recognition task, which is a largely unexplored area, and create a new dataset within the domain. The AlexNet architecture was noted for its significant breakthrough in machine learning and computer vision-related tasks. Since then, newer architectures have been introduced, such as Inception V3, Inception Resnet V3, Xception, and more. The objective of this research study was to make distinctions between the different types of backlift techniques in cricket. Having discussed the results proposed by each architecture, the aim of this approach has been achieved. With the Xception architecture performing optimally, it would be useful further to investigate other biomechanical networks in future sports science research. This study also provides a way forward in the automatic recognition of player patterns and motion capture, making it less challenging for sports scientists, biomechanists, and video analysts working in the field. Future improvements can be made by investigating more finer grade movements by looking at segmenting specific objects using semantic segmentation methods^36,37^ and factoring more temporal aspects such as finer grain gestures^38^. Further investigation is required to determine how a model should be trained to maximise the benefit for both coaches and athletes for all sports. Furthermore, future work should evaluate the generalisation ability of similar models in a match situation and analyse players of varied demographics, including age, gender, skill level and format type. The models could provide better correlations to batting backlift technique as well as cricket batting performance.
